# Managing and mitigating fatigue in the era of changing resident duty hours

**DOI:** 10.1186/1472-6920-14-S1-S3

**Published:** 2014-12-11

**Authors:** Derek Puddester

**Affiliations:** 1 University of Ottawa, Ottawa, Ontario, Canada

## Abstract

The medical establishment is grappling with the complex issue of duty hour regulations – an issue that is a natural consequence of the numerous changes in medical culture and practice that have occurred over the course of decades. Sleep deprivation resulting from long duty hours has a recognized impact on resident health and wellness. This paper will briefly outline the evolution of the concept of well-being in residency, review the specific theme of fatigue management within that context, and describe strategies that may be used to mitigate and manage fatigue, as well as approaches that may be taken to adapt to new scheduling models such as night float. Finally, the paper will call for a change in the culture in our workplaces and among our residents and faculty to one that promotes good health and ensures that we maintain a fit and sustainable medical workforce.

## Introduction

The landscape of medicine in the 1970s that was described in House of God [1] seems bizarre to us today – an odd mix of immaturity, misogyny, narcissism, and incredible self-sacrifice. Over the past several decades, with the skill of thoughtful and courageous leaders and the ongoing advocacy of residents, medicine has tackled critical issues in medical education and training, creating global renewal in curricula, evaluation, and social engagement. Medicine has also shone a light inward and renewed its interest in and commitment to professionalism. Finally, in recent years, the profession has bravely tackled the challenging themes of intimidation and harassment, equity, workplace safety, and physician health and well-being. It would be naive to suggest that medical culture still does not have areas upon which it needs to continue to reflect deeply; however, it is reasonable to suggest that the profession has matured and that it now has an ability to reflect that is in keeping with its age.

There has been increasing evidence that physicians experience symptoms of burnout – one component of overall physician health – not only at a high incidence and prevalence, but also at rates significantly higher than in the general population [2-6]. Burnout – characterized by a loss of interest and enthusiasm in work, feelings of deep cynicism and depersonalization, and an increasing sense of lack of efficacy – can have devastating consequences. Medicine takes burnout seriously. It is associated with preventable consequences, both professional (e.g., medical errors, lower quality of care, disruptive behaviour) [7,8] and personal (e.g., substance abuse, relationship strain and fracture, mental health disorders) [9].

Specifically, over the past few decades, resident organizations in Canada and elsewhere have advocated for increased attention to the health and wellness of health professionals. In the late 1990s, the Canadian Federation of Medical Students (CFMS) and the Canadian Association of Internes and Residents (CAIR) published the world’s first position papers on physician health and well-being within months of each other. Shortly thereafter, the Canadian Medical Association released its own policy [10]. In addition, the Canadian Patient Safety Institute (CPSI) has called for deliberate attention to the human and environmental factors related to patient safety. Governments have also begun to demonstrate an increased interest in health-professional health and sustainability, and education and training leaders (such as the Royal College of Physicians and Surgeons of Canada) have developed educational products and services to assist in teaching and evaluating the competency of professionalism within the CanMEDs framework [11]. While all of this was occurring in Canada, similar efforts were being made in other countries around the world.

Within this growing global context, a natural step forward is an increased attention to safety issues for patients, families, and communities. Duty hour regulation is a natural part of this evolution; the discourse is necessary, and the innovation that is emerging is to be expected.

There is little disagreement that the medical trainees of yesterday and today have experienced sleep deprivation as part of their education and training. Many factors contribute to this: the high volume of material to be mastered, an endless amount of clinical service to be offered, and much life to be lived. Historically, fatigue management has had a low profile within and little relevance to the medical profession. In addition, issues of personal vulnerability were not readily embraced, perhaps for fear of bringing shame to the profession.

History is full of critical moments that should be remembered. One such moment occurred on March 5, 1984 when 19-year-old Libby Zion died from serotonin syndrome at New York Hospital while in the care of an attending physician and two resident physicians. Zion’s family were strong advocates for the reform of duty hour regulations, and Sidney Zion, Libby’s father, wrote in an op-ed originally published in The New York Times: "You don't need kindergarten to know that a resident working a 36-hour shift is in no condition to make any kind of judgment call—forget about life-and-death” [12]. This tragedy triggered a number of investigations, reviews, and studies, and, in the years that followed, laws were enacted in the United States to address resident duty hours and standards of clinical supervision.

Over the past two decades, duty hours have become a globally relevant issue. In the United States, the Accreditation Council for Graduate Medical Education (ACGME) restricts weekly resident duty hours to 80 (including call), with a maximum duty period of 16 hours for first-year residents [13]. Among European Union countries, the Working Time Directive restricts duty hours for trainees to 48 per week, with a maximum duty period of 13 continuous hours [14]. In Canada, while there is no national policy for duty hour restrictions, a Quebec arbitration ruling that sets duty hours at 16 per day and further stipulates that 24-hour call duty violates the provincial and national charters of rights and freedoms is currently being evaluated [15].

## Fatigue management

Strong and consistent data show that sleep-deprived trainees are at risk of being injured in the workplace (e.g., needle stick injury, exposure to contaminated bodily fluids) and making errors related to their patients’ care (e.g., adverse events, prescription errors, misinterpretation of laboratory data) [16], in addition to other personal consequences such as relationship stress and mood disorders [17,18]. Performance-related effects include impaired memory, compromised problem-solving abilities, impaired fine-motor skills, and reduction in quality of teaching to junior learners [17,19-21]. In addition, several studies have demonstrated that residents have an alarmingly high rate of motor vehicle accidents and other traffic incidents after a period of sleep deprivation [22].

As the profession has evaluated the data related to fatigue management, it has been noted that few physicians are well educated in sleep physiology and pathology. As a result, they tend not to know how best to mitigate and manage fatigue and can exhibit cognitive distortions about their own levels of alertness and sedation. Indeed, resident physicians are subject to a number of determinants of fatigue such as phase of life issues (e.g., socializing, parenting, studying), work issues (e.g., being on call with less than maximum recovery), other health issues (e.g., stress, depression, burnout, sleep apnea), and nutritional/chemical issues (e.g., use of caffeine, lack of healthy food and exercise).

One particular aspect of sleep physiology of relevance to this discussion is sleep inertia (a period of mild cognitive impairment and motor dysfunction that occurs when a trainee wakes from non-rapid eye movement (NREM) sleep, typically in the middle of the night or when sleeping after a period of sleep deprivation). This period of impairment can last between 10 and 120 minutes. Trainees should be mindful of this, and should be particularly careful of the decisions made and actions taken during this time, as there is evidence that this period is associated with an increased risk of trainee error. The risk can be minimized by light exposure, physical activity, frequent napping, and appropriate nutrition and hydration.

Managing fatigue is a skill that is best taught by experts with appropriate evaluation and revision to ensure maximum competency. Studies that have evaluated how trainees managed sleep de novo have found high rates of behaviour that, while adaptive in the short term, have associated risks and negative outcomes about which few trainees are aware [23].

Perhaps the most obvious and challenging intervention is the one that is most needed: to create a workplace and training environment that encourages and promotes good health, including good sleep. This requires that both trainees and supervisors are fully educated on duty hour standards and that there is a cultural expectation that these standards will be fully enforced. In addition, there is a need to train residents in self-assessment and self-awareness skills to empower them to identify when they might be at an increased risk of error. For some institutions, this will trigger a number of cultural changes as well as very practical human resource issues. Regardless, it is essential that legislative and negotiated duty hours be respected by all; this is one way in which the medical establishment can demonstrate its professionalism.

## Strategies to manage and mitigate fatigue

### Pre-call strategies

Programs should offer trainees formal education in fatigue management, perhaps starting with basic concepts of sleep hygiene. For example, knowing the skills required to help trainees minimize the amount of sleep debt they carry into a period of call can have a significant impact on their risk of sleep deprivation. This may require formal training in preventive health that focuses on areas such as the appropriate minimum levels of physical activity needed, daily sleep requirements, healthy nutrition, and stress and time management.

It can also be valuable for trainees to access a primary care provider on a regular basis who can screen them for health issues that may pose a threat to fatigue management (e.g., mood disorders, endocrine disorders, sleep apnea, substance use or abuse disorders) and offer the appropriate treatment.

If possible, trainees should strive to have a short nap between the end of daytime duties and initial on-call handover. There is ample evidence that even a short nap of 15 to 30 minutes can improve an individual’s alertness. A nap between 2:00 p.m. and 5:00 p.m. generally aligns with circadian rhythms and provides a boost in alertness and cognition. In general, longer naps are not advised as they can contribute to sleep inertia, which can impair performance.

### On-call strategies

As mentioned earlier, even short naps (e.g., 15 minutes) can contribute to a reduction in the negative effects of sleep disruption. Naps can be facilitated by call rooms that are kept dark, cool, and as free from external noxious stimuli as possible [24].

Appropriate caloric intake is essential given the additional calories burned while working intensely. There is evidence that dehydration and reduced glucose levels can lead to basic physiologic deficits and can have a negative impact on decision making [25]. Hospitals often do not provide on-call trainees with access to fresh and nutritious food, and the standards of hospital cafeterias and vending machines vary widely. Should hospitals not provide such services to trainees, programs should encourage residents to bring their own food to work. For some trainees, this may be best facilitated by offering access to nutritional training/advice (e.g., learning how to pre-prepare and bring their own meals and snacks for an on-call period).

Given the physical exertion involved on-call, many trainees experience symptoms of dehydration, including fatigue. Having an easily accessible supply of water can eliminate the risk of dehydration and contribute to alertness, mood stability, and comfort. Water that is naturally flavoured with fresh fruit is preferable to water that is flavoured with chemicals or concentrated artificial sugars (including sodas), as these can contribute to brief peaks and lows in blood sugar as well as to diuresis.

Caffeine can be of value on-call but its use needs to be considered within the context of other physiological issues. In general, caffeine has a rapid onset of action and can last for up to seven hours in a typical young adult. As such, it can interfere with post-call sleep, so its use later in the call period should be discouraged. In addition, individuals can develop a tolerance to caffeine, which can lead to the need for higher and more frequent doses of caffeine to achieve similar physiological effects. This can create a new challenge for trainees as they may need to manage post-call symptoms arising from the discontinued use of caffeine (e.g., headache, dizziness, malaise). Finally, flavouring coffee with sugar and milk products can add a source of unhealthy calories that may contribute to weight gain.

Finally, where possible, short breaks in natural light for fresh air and meditation can allow for physiological and psychological refreshment.

### Post-call strategies

Post-call handover is now recognized as a clinical skill. Trainees and supervisors should bear in mind that handover typically occurs when the body is least able to manage the negative effects of sleep deprivation (i.e., early morning). As such, creating standardized approaches to handover can be helpful in promoting the safety and quality of care. This will also allow trainees to exit the hospital in a timely fashion and will allow the day team to assume their duties. In addition, trainees should prearrange transport home, preferably taking public or shared transportation rather than driving themselves. Signs of sleepiness while driving include lane drifting, difficulty focusing, missing turns, nodding, and closing eyes at stoplights. There are no environmental modifications (e.g., loud music, blasts of wind, pain, stimulant use) that effectively manage sleep impairment on the road. Simply put, if a trainee experiences fatigue while driving he or she should pull over and take a short nap (15 to 20 minutes) or should arrange for other transportation home.

Once home, consideration to nutrition is essential. Residents should ensure that they rehydrate as much as possible, have a light breakfast, minimize their use of caffeine, and ensure that external distractions that may awaken them (e.g., smart phone alerts, telephone calls, doorbells, pagers) are managed. Some physicians find that using white noise machines or low decibel music can help block out potential sounds that may awaken them. Others have found that ear plugs and eye shades facilitate solid sleep.

In general, it is advisable to defer exercise until after sleep, as heavy exercise can contribute to difficulty sleeping and there may also be an increased risk of injury if one exercises while in a sleep-deprived state.

The use of pharmaceuticals and chemicals to assist in post-call sleep restoration is an area that has been inadequately studied. For example, melatonin is widely used as a natural hypnotic; however, its use in specific situations, such as residency, has not been well studied. Hypnotics may be of short-term value in particular cases where sleep onset is impaired, but they should only be prescribed by physicians who have had training in occupational medicine or physician health and who understand the unique workplace issues experienced by physicians. Alcohol is of little value; while it may induce the onset of sleep, it can damage normal sleep architecture, and may contribute to a sleep deficit.

Programs and trainees should be reminded that their sleep deficit from call cannot be readily managed in a single night. Generally, physicians require two full nights to recover from one night of sleep impairment. It is important to build this into the overall culture and academic structure of programs. As such, the scheduling of academic activities on post-call days will need to be curtailed or mindfully rescheduled. The responsibility for thoughtful scheduling should be shared between program directors and residents.

**Figure 1:**
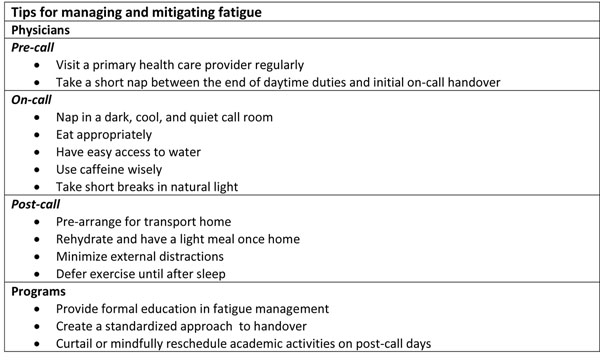
Tips for managing and mitigating fatigue

Figure 1 contains tips for managing and mitigating fatigue.

### Night float

Increasingly, programs are experimenting with the concept of a night float to assist in meeting both patient care and human resource needs. The use of a night float can be helpful; however, it does create some potential challenges for those who are adjusting to working hours that are different from their baseline standard [26]. It generally takes about a week for the body to fully adjust to an ongoing shift in sleep hours. It is beneficial to be mindful of this fact and to employ the following strategies:

• Acknowledge that adjusting to night shifts takes time and may cause both physical (e.g., fatigue, lethargy) and psychological (e.g., irritability, decreased cognitive functioning) symptoms. It is wise to alert family and friends of the shift change so that their expectations are reasonable.

• Trainees should establish a new period of sleep that matches their physiological needs (i.e., seven to nine hours). For some, this will require significant changes to their personal and recreational activities and routines. It is reasonable to recruit and involve ones friends and families in this shift and to develop a clear understanding of why and how this new sleep period should be protected.

• Light exposure is important and needs to be optimized thoughtfully. Having access to natural bright light can help to restore or maintain alertness. However, exposure to bright light after a night shift should be minimized. Wearing sunglasses, ensuring rooms are dark and cool, and minimizing outdoor activity can assist with sleep promotion.

• Trainees should maximize their resiliency by attending to healthy nutrition, exercise, recreation, and social engagement as best as they can. This may be best facilitated by offering both formal and informal curricula to help trainees, and groups of trainees, to nurture and enhance these skills.

### If things become problematic

From time to time, it is not uncommon for some trainees to struggle with fatigue to the point of needing assistance. It is professional and healthy to both acknowledge such a need and to seek consultation early. In Canada, trainees can contact their personal physician, their university wellness program, their residency association, or their provincial physician health program, or they can seek help online (e.g., www.ephysicianhealth.com) or consult published resources such as the CanMEDS Physician Health Guide [27]. It may be appropriate to consider referral to a sleep specialist and/or an occupational health specialist should fatigue management become a clinical issue that threatens a trainee’s normal career trajectory. Finally, it is essential that trainees not be tempted to self-prescribe or to prescribe medications for other trainees, as this may serve to create ethical, professional, and legal problems.

### The role of advocacy and influence

Fostering change in any community, including a community of practice as ancient and complex as medicine, is both exciting and challenging. In their book entitled Influencer: The Power to Change Anything, Patterson and colleagues highlight a strategic framework that can be very helpful for fostering and sustaining change for health professionals [28]. First, they examine influence challenges to determine what resistance might be due to a lack of motivation and what may be due to a lack of skill. Second, they consider personal factors (e.g., lack of insight would be a personal motivation problem, while an inability to define the safe use of caffeine would be a personal ability issue), social factors (e.g., peer pressure, the most significant source of influence, is a social motivational tool), and structural factors (e.g., lack of institutional policies and procedures might reflect a structural ability problem). The influencer model can help programs conceptualize ways to consider the factors involved in change management.

Peer pressure – a form of social motivation – is a particularly relevant determinant of change. Residents in Canada have gone to court, written national position papers, and developed negotiated employment agreements that create a shared community of expectation regarding well-being and fatigue management. Change has already come from within that particular part of our community of practice. However, other cohorts in the community (e.g., more senior generations of physicians) may not share the same value set, and intergenerational tensions may evolve and will need to be addressed in a respectful manner.

Policies and procedures – a form of structural ability – are also a strong determinant of change. As hospitals and universities develop and implement policies and procedures to assist in the implementation of duty hour restrictions, changes will become easier to implement. Other structural abilities, such as having adequate clinical personnel to meet clinical demands, need to be managed with thoughtfulness and innovation.

Knowledge of sleep physiology and pathophysiology – both personal and structural abilities – may need to be addressed in the curriculum and through evaluation. Poor personal motivation, largely arising from disparate value sets, apathy, fear, or other relevant issues, may also need to be addressed.

Regardless of these and other challenges, important influence is coming from patients, their families, other health professionals, and funders of the health care system. Given the risks inherent in fatigue, the motivation for system-level change is remarkably high.

## Conclusion

The contemporary landscape of medicine is addressing duty hour regulation and fatigue management at an appropriate time in its development. As the profession strives to develop and implement new strategies to promote patient care and safety, issues of personal health and sustainability will need to be thoughtfully and carefully addressed. Changes to curriculum, program design, clinical services, and evaluation will be necessary, and this change will require leadership and influence if it is to be successful across the profession.

## Competing interests

The author declares that they have no competing interests.

## Declarations

Resources and secretariat support for this project was provided by the Royal College.

This article has been published as part of *BMC Medical Education* Volume 14 Supplement 1, 2014: Resident duty hours across borders: an international perspective. The full contents of the supplement are available online at http://www.biomedcentral.com/bmcmededuc/supplements/14/S1. This article was submitted and peer reviewed in 2012. Final acceptance for publication as part of this supplement was in 2014.

Publication of this supplement was supported and funded by the Royal College of Physicians and Surgeons of Canada. The funding agency played no role in the design, in the collection, analysis and interpretation of data; in the writing of the manuscripts; and in the decision to submit the manuscript for publication. The articles have been through the journal's standard peer review process for supplements. The Supplement Editors declare that they have no competing interests.
